# From Gamma Rays to Green Light: Comparative Efficacy of Indocyanine Green and Technetium-99m in Sentinel Lymph Node Biopsy for Breast Cancer

**DOI:** 10.3390/medsci13040231

**Published:** 2025-10-13

**Authors:** Vlad Alexandru Gâta, Radu Alexandru Ilieș, Nicoleta Zenovia Antone, Roxana Pintican, Codruț Cosmin Nistor-Ciurba, Ștefan Țîțu, Alex Victor Orădan, Maximilian Vlad Muntean, Gheorghe Gerald Filip, Alexandru Irimie, Patriciu Andrei Achimaș-Cadariu

**Affiliations:** 1 Department of Oncological Surgery, “Iuliu Hațieganu” University of Medicine and Pharmacy, 400012 Cluj-Napoca, Romania; 2 Department of Oncological Surgery and Gynecological Oncology, “Prof. Dr. Ion Chiricuță” Institute of Oncology, 400015 Cluj-Napoca, Romania; 3 Faculty of Medicine, “Iuliu Hațieganu” University of Medicine and Pharmacy, 400012 Cluj-Napoca, Romania; 4 Department of Oncology, “Prof. Dr. Ion Chiricuță” Institute of Oncology, 400015 Cluj-Napoca, Romania; 5 Department of Radiology, “Iuliu Hațieganu” University of Medicine and Pharmacy, 400012 Cluj-Napoca, Romania; 6 Department of Plastic Surgery, “Prof. Dr. Ion Chiricuță” Institute of Oncology, 400015 Cluj-Napoca, Romania; 7 Department of Plastic and Reconstructive Surgery, “Iuliu Hațieganu” University of Medicine and Pharmacy, 400012 Cluj-Napoca, Romania; 8 Department of Surgery, Ponderas Academic Hospital, 014142 Bucharest, Romania

**Keywords:** breast cancer, sentinel lymph node biopsy, Technetium-99m, Indocyanine Green, axillary staging, surgical margins

## Abstract

**Background/Objectives**: Sentinel lymph node biopsy (SLNB) is currently the standard approach for axillary staging in breast cancer. Conventional techniques are radioisotope-based (Technetium-99m, Tc99m) and remain widely used, but novel tracers like Indocyanine Green (ICG) fluorescence provide potential advantages regarding feasibility and logistics. **Methods**: We conducted a prospective, observational study including 476 female patients diagnosed with primary invasive breast cancer who underwent SLNB at the Institute of Oncology “Prof. Dr. I. Chiricuță”, Cluj-Napoca, Romania, between January 2022 and May 2025. Clinical, surgical, and pathological variables were systematically extracted. SLNB was performed using either Tc99m or ICG, according to institutional protocols. Comparative analyses were performed to evaluate sentinel node characteristics, histopathological parameters, and positive surgical margins predictors. **Results**: The median age was 60 years (IQR: 52–69). Breast-conserving surgery (BCS) was performed in 77.9% of cases, while mastectomy was performed in 22.1%. Sentinel lymph node positivity was reported in 25.6% of cases, with no significant differences in the number of excised or metastatic nodes between Tc99m and ICG (mean nodes: 3.23 vs. 3.20, *p* = 0.860; mean positive nodes: 0.35 vs. 0.36, *p* = 0.897). Histologically, invasive carcinoma NST was predominant (90.1%), and surgical margins were negative in 96.8% of patients, with all margin-positive cases occurring following BCS. No pathological markers (grade, Ki67, TILs, DCIS extent) predicted margin status or nodal involvement. Notably, younger age correlated inversely with the extent of ductal carcinoma in situ (r = −0.21, *p* < 0.00001). **Conclusions**: Tc99m and ICG provided comparable diagnostic performance in performing SLNB, with equivalent rates of nodal detection and pathological yield. These findings support that ICG is a safe and effective alternative for routine axillary staging in breast cancer.

## 1. Introduction

Introduced in the 1990s, sentinel lymph node biopsy (SLNB) quickly became the standard for axillary staging in breast cancer thanks to its increased accuracy and reduced morbidity compared to traditional approaches (axillary lymph node dissection) [1,2]. Randomized trials demonstrated that SLNB provides equivalent information on axillary status, with no differences in regional control, disease-free survival, or overall survival in clinically node-negative patients [2].

Initially applied to invasive cancer, its applications have spread to ductal carcinoma in situ, recurrence of the disease, respectively, for patients receiving neoadjuvant therapy. There are numerous advantages of this technique, including refined histologic assessment with multiple sectioning and immunostains, as well as a reduction of lymphedema [1,2].

According to the American Society of Breast Surgeons, SLNB can be skipped in patients over 70 years of age who present with clinically T1–T2N0, hormone receptor–positive, HER2-negative breast cancer [3,4]. Recently, SLNB has also been adopted for patients who were initially diagnosed with node-positive disease and who convert to clinically node-negative status following neoadjuvant chemotherapy [4,5]. Hence, the role of SLNB has extended beyond its initial application in cases with clinically node-negative early breast cancer. While approximately 75% of SLNBs are negative, current research relies on selecting patients with very low chances of axillary metastasis who might safely avoid even SLNB [2,6].

Notably, SLNB is associated with increased quality of life and decreased morbidity when compared to axillary lymphadenectomy, setting it as the standard of care for axillary staging in early breast cancer [2,6].

The classical technique for performing SLNB has relied on a combination of radioisotopes (RI) (introduced by Krag et al., 1993 [7]) and blue dye (BD) (Giuliano et al., 1994 [8]). Sentinel lymph node detection implies tracers, but current standards have their own limitations. RI sometimes faces logistical barriers, while BD represents the only option in some countries, with a small risk of developing allergic reactions, and depending mainly on surgical experience. These drawbacks encouraged the development of alternatives, such as Indocyanine Green (ICG) and superparamagnetic iron oxide (SPIO)—showing promising results in early breast cancer [2,9,10].

ICG represents a water-soluble substance with near-infrared fluorescence, which was approved by the US Food and Drug Administration to be applied in some circumstances, such as measurement of cardiac output, assessment of liver function, and evaluation of ocular perfusion. Its absorption peak is around 800 nm and emits fluorescence near 830 nm. After being injected into the dermis, ICG allows intraoperative SLN mapping via a laser-based imaging system that provides real-time lymphangiography. The technique is based on the fluorescent signal, which is generated by the binding properties of ICG to plasmatic proteins within the lymphatic vessels [11,12].

Fluorescence-guided SLNB using ICG offers a wide variety of benefits, including accurate localization of the site of incision, direct visualization of lymphatic drainage toward the axillary lymph nodes, increased detection rates of sentinel nodes, and a low risk of developing complications. Its strong resemblance to methods based on radioisotopes further supports the oncological safety of this approach [12,13].

Latest research underscores that ICG consistently outperforms BD when used alone for SLNs detection, returning higher identification rates and fewer false-negative results [4]. Furthermore, studies demonstrate that the accuracy of ICG is at least comparable to that of RI, both as a single tracer and when evaluated against the conventional dual-tracer approach (combining RI and BD) [14,15,16]. This growing body of evidence supports that ICG is a reliable and effective alternative, raising the question of whether it could complement (or even replace) traditional tracers in routine clinical practice.

The main objective of the current study is the comparison between the two tracers: ICG and Tc99m, for performing SLNB in patients diagnosed with breast cancer (primary tumor). In addition to tracer comparison, margin status was analyzed as a secondary exploratory endpoint, given its clinical relevance in breast-conserving surgery. A secondary objective of this study was the analysis of the broader histopathological and surgical characteristics of our cohort, focusing on oncological outcomes. Ultimately, this study aimed to provide clinically relevant evidence regarding the comparison between the use of ICG and Tc99m.

## 2. Materials and Methods

The current study was approved by the Ethics Committee of the Institute of Oncology “Prof. Dr. Ion Chiricuță”, Cluj-Napoca, Romania (approval no. 308/17 December 2021). This prospective observational comparative study included patients who were diagnosed with primary breast cancer, undergoing sentinel lymph node biopsy (SLNB), between 1 January 2022 and 30 May 2025 at the Institute of Oncology “Prof. Dr. I. Chiricuță” (IOCN), Cluj-Napoca, Romania. Relevant clinical, imaging, and pathological data were systematically recorded (following each surgical case), and a database was created for the current study. The prospective design of our study allowed a standardized collection of the data, ensuring consistency across cases.

Practically, the acquisition of data included obtaining informed consent from all participants preoperatively. Data collection was realized immediately postoperatively by the surgical team, with subsequent verification for accuracy and completeness. Regarding the surgical procedure, it required strict adherence to the institutional protocol on SLNB. From an ethical perspective, the current research was conducted under the approval of the institutional review board and ethics committee, and patient confidentiality was ensured, in compliance with national and European data protection regulations (GDPR).

### 2.1. Study Population

A total of 476 female patients were included, based on the following inclusion criteria:

- primary invasive breast cancer that was confirmed histologically,
- SLNB performed using either Technetium-99m (Tc99m) or Indocyanine Green (ICG) as a tracer,
- availability of complete surgical and histopathological data.

Patients who did not undergo SLNB, those who received alternative methods of performing SLNB, or patients who had incomplete data regarding nodal status or surgical margins were excluded from our analysis.

### 2.2. Data Collection

Relevant clinical, surgical, and pathological data were extracted from the abovementioned database (and anonymized prior to analysis). The dataset included:

- demographic data (age, neoadjuvant treatment),
- surgical data (type of procedure: breast-conserving surgery or mastectomy),
- tumor staging (clinical T and N stage),
- SLNB characteristics (number of sentinel nodes removed, number of positive nodes, type of tracer used),
- detailed histopathological parameters: histological subtype, tumor grade (according to Nottingham grading system), Ki67 proliferation index, tumor-infiltrating lymphocytes (TILs), presence of ductal carcinoma in situ (DCIS), and presence of lymphatic, vascular, or perineural invasion.

Surgical margins status (R0: negative vs. R1: positive) based on histopathological exam of the resected specimen.

### 2.3. Sentinel Lymph Node Identification Technique

SLNB was performed either using Indocyanine Green (ICG) injected periareolar subdermal and detected using near-infrared fluorescence imaging, or Technetium-99m nanocolloid particles injected in a similar way and detected using a gamma probe. The choice of tracer was not randomized, being based primarily on the existing institutional protocols and availability.

For patients undergoing ICG-guided SLNB, Indocyanine Green (Verdye 5 mg/mL) was diluted to a final concentration of 0.25 mg/mL and injected into the subdermal periareolar region in two separate points, irrespective of tumor location. Injections were performed after induction of anesthesia, and SLNB was always performed prior to lumpectomy. The sentinel lymph nodes were identified intraoperatively using the Artemis Surgical Device or the Pinpoint Striker Device.

For Tc99m-guided SLNB, the radiotracer was injected into the subdermal periareolar region (single injection point) in the Department of Nuclear Medicine, according to a same-day injection protocol. Cutaneous marking was performed, and intraoperative localization was achieved using the Europrobe gamma detection system. Nodes were harvested if they demonstrated at least 10% of the maximum injection site signal, measured both in vivo and ex vivo.

SLNB was indicated only for patients with early-stage breast cancer and a clinically node-negative axilla (N0). All cases were discussed and approved in a multidisciplinary tumor board (MDT). The inclusion was irrespective of tumor characteristics, as long as patients met the standard guideline criteria for SLNB in early breast cancer.

### 2.4. Statistical Analysis

Descriptive statistics were used for the characterization of the cohort. Continuous variables were expressed as mean ± standard deviation (SD) or median with interquartile range (IQR), in each case. Categorical variables were represented by absolute and relative frequencies. Comparisons between separate groups were performed using the independent *t*-test for continuous variables and the Chi-squared test or Fisher’s exact test for categorical variables. Pearson correlation coefficients were calculated to explore the relation between continuous histopathological parameters and clinical variables.

In addition, an exploratory multivariable logistic regression was performed to evaluate independent predictors of SLN positivity. The model included age (continuous), clinical T stage, tumor grade, neoadjuvant therapy (yes/no), and type of surgery (BCS vs. mastectomy). Odds ratios (OR) with 95% confidence intervals (CI) were reported, and *p* < 0.05 was considered statistically significant.

All statistical analyses were performed using Microsoft Excel, Microsoft 365 (Version 2407), and IBM SPSS Statistics for Windows (Version 29.0).

## 3. Results

### 3.1. Patient Demographics and Surgical Treatment

In our cohort, the median age at diagnosis was 60 years (interquartile range, IQR: 52–69 years), respectively, a mean of 59.4 years (standard deviation, SD: 11.0; range: 25–83). Most of the patients (*n* = 346, 72.7%) did not receive neoadjuvant systemic treatment, whereas the other 130 (27.3%) underwent neoadjuvant therapy.

**Figure 1:**
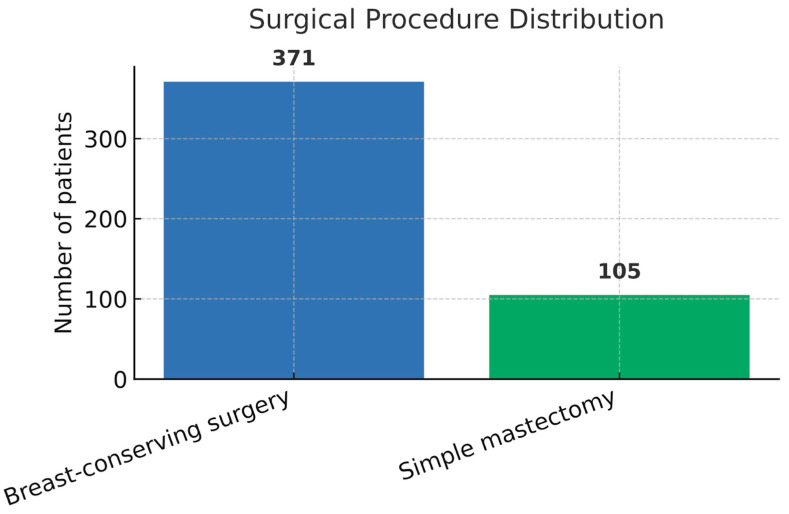
Surgical procedure distribution. Bar chart showing the distribution of breast-conserving surgery (*n* = 371, 77.9%) versus simple mastectomy (*n* = 105, 22.1%) among the study cohort (*n* = 476).

Breast-conserving surgery (BCS) was performed in 371 patients (77.9%), while simple mastectomy was performed in 105 cases (22.1%). In addition, the mean age of patients who underwent BCS was 60.2 years (SD: 11.3), respectively, 58.2 years (SD: 10.5) for those who received mastectomy, with no statistically significant difference between the two groups (*p* = 0.054, independent samples *t*-test). A detailed representation of demographic and surgical characteristics is presented in Table 1 and Figure 1.

### 3.2. Tumor Staging and Sentinel Node Findings

**Figure 2:**
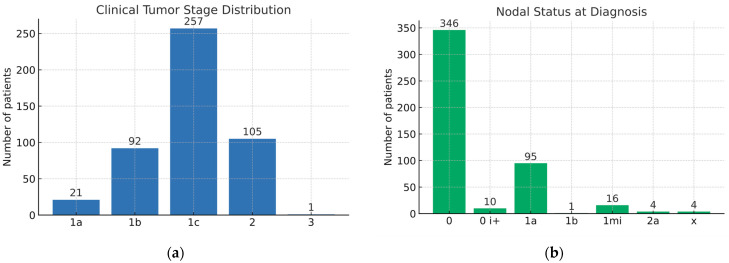
(**a**) Bar chart illustrating the distribution of patients according to clinical T stage; (**b**) Bar chart representing nodal status (N stage) at diagnosis.

In our cohort, the clinical tumor stage (T) distribution was as follows: T1c in 257 patients (54.0%), T1b in 92 (19.3%), T2 in 105 (22.1%), T1a in 21 (4.4%), and T3 in 1 case (0.2%), as shown in Figure 2a.

Regarding nodal status (N), most patients were N0 (*n* = 346, 72.7%). Other stages included N1a in 95 patients (20.0%), N1mi in 16 (3.4%), N2a in 4 (0.8%), and N1b in 1 case (0.2%). Additionally, isolated tumor cells (ITC, N0, i+) were identified in 10 patients (2.1%), while nodal status was undetermined (Nx) in 4 cases (0.8%), as indicated in Figure 2b.

The average number of sentinel lymph nodes excised was 3.22 (SD: 1.98), with a median of 3 (range: 1–11). The average number of positive sentinel nodes was 0.35 (SD: 0.73), with a median of 0 (range: 0–5). In total, 122 patients (25.6%) had at least one positive sentinel lymph node.

SLNB was performed using either Technetium-99m (Tc99m) in 286 cases (60.1%) or Indocyanine Green (ICG) in 190 cases (39.9%). There were no statistically significant differences between the two tracer groups regarding the number of sentinel lymph nodes excised (mean Tc99m: 3.23 vs. ICG: 3.20; *p* = 0.860) or the number of positive nodes (mean Tc99m: 0.35 vs. ICG: 0.36; *p* = 0.897), as represented in Table 2. The rates of lymphatic invasion were 17.5% in the Tc99m group and 14.2% in the ICG group (*p* = 0.411), while perineural invasion was present in 15.4% and 16.8%, respectively (*p* = 0.766). Vascular invasion was rare in both groups (Tc99m 1.05% vs. ICG 0.53%), with no statistically significant difference (*p* = 0.921).

### 3.3. Histopathological Features

Detailed histological characteristics are summarized in Table 3. The most frequent histological subtype in the cohort was represented by invasive carcinoma of no special type (NST), being identified in 429 cases (90.1%). Other subtypes included invasive lobular carcinoma (*n* = 11, 2.3%), mixed NST with micropapillary features (*n* = 19, 4.0%), NST with mucinous features (*n* = 10, 2.1%), and NST with cribriform features (*n* = 7, 1.5%).

According to the Nottingham system, grading revealed 172 cases (36.1%) with grade 1 tumors, 250 (52.5%) with grade 2, and 54 (11.3%) with grade 3. The mean Ki67 proliferation index was 17.7% (SD: 11.9%), with a median of 15% (IQR: 10–25%). Tumor-infiltrating lymphocytes (TILs) were represented with a mean of 7.5% (SD: 10.9%), respectively, a median of 5% (IQR: 1–10%). Associated ductal carcinoma in situ (DCIS) was present in 78.2% of cases, with a mean component of 11.6% (SD: 17.1%).

**Figure 3:**
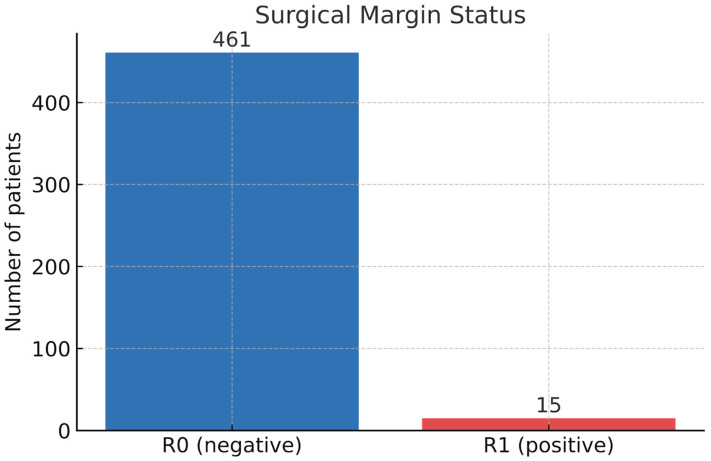
Bar chart showing the status of surgical margins. Negative margins (R0) were achieved in 96.8% of patients (*n* = 461), while positive margins (R1) were identified in 3.2% (*n* = 15).

In terms of surgical margins status, negative margins (R0) were reported in 461 patients (96.8%), while the remaining 15 patients (3.2%) had positive margins (R1), as shown in Figure 3. Margin status was not significantly associated with the molecular subtype (*p* = 0.75, Chi-squared test).

### 3.4. Associations Between Pathological Parameters and Sentinel Node Positivity

Among the 122 patients (25.6%) who had at least one positive sentinel node, the mean Ki67 was 18.0% (SD: 11.9%), compared to 17.7% (SD: 11.9%) in node-negative cases (*p* = 0.797). Also, TIL levels were 7.2% vs. 7.6% (*p* = 0.749), and there was no statistically significant difference regarding the Nottingham grade distribution (mean grade 1.80 vs. 1.74; *p* = 0.380).

To further explore predictors of sentinel lymph node positivity, a multivariable logistic regression model was constructed (presented in Table 4). Lymphovascular invasion emerged as the only independent predictor of nodal involvement (OR = 3.42; 95% CI: 2.01–5.83; *p* < 0.001). Age, tumor grade, neoadjuvant therapy, and type of surgery did not reach statistical significance.

### 3.5. Predictors of Positive Surgical Margins

In the cohort, a total of 15 cases (3.2%) presented positive surgical margins (R1) following breast surgery. In an analysis of potential risk factors, we investigated whether histopathological features could predict margin status.

No statistically significant association was recorded between margin positivity and the following: presence of ductal carcinoma in situ (mean DCIS component 18.1% in R1 vs. 11.4% in R0, *p* = 0.341), Ki67 index (*p* = 0.918), tumor grade (*p* = 0.876), or presence of lymphovascular invasion (*p* = 0.958). Furthermore, the distribution of each histological subtype did not differ significantly between the R0 and R1 groups (*p* = 0.87).

The type of surgical intervention in relation to the status of surgical margins did not show statistical significance (*p* = 0.076). All cases with positive margins were registered after BCS.

### 3.6. Association Between Age and DCIS Component

Interestingly, an inverse correlation was recorded between the age of patients and the extent of associated ductal carcinoma in situ (DCIS) (r = −0.21, *p* < 0.00001, Pearson correlation test). Younger patients tended to present more commonly with tumors that were characterized by a significant in situ component (even though the lesion was invasive).

## 4. Discussion

### 4.1. Interpretation of the Main Results

In our cohort of 476 breast cancer patients who underwent SLNB, we identified a few clinically relevant associations and trends, while also uncovering a potential relation between age and the development of in situ disease.

To begin with, the rate of sentinel node positivity was 25.6%. A few classical markers, like tumor grade, Ki67 index, and TILs, did not significantly differ between the groups of node-negative and node-positive patients. These findings might highlight the multifactorial character of nodal metastasis and support that such markers are insufficient predictors, taken separately. Despite this, lymphatic invasion was met more frequently in node-positive patients, suggestive of tumor spread via lymphatic channels.

Regarding resection margins, only 3.2% of patients presented positive surgical margins (R1). Even if DCIS was present in 79.0% of the cohort, no statistically significant relation was found between the extent of in situ disease and the presence of R1 margins. In our cohort, all cases with positive margins were observed after BCS. Although this association showed a numerical trend (*p* = 0.076), it did not reach statistical significance at the predefined threshold. This finding suggests that margin positivity is more likely related to surgical technique and anatomical factors than to histopathological characteristics. Larger studies are needed to clarify whether this trend holds true in broader patient populations.

Surprisingly, in our cohort, patients who underwent mastectomy were slightly younger compared to those who were treated with BCS. This apparent discrepancy may be explained by some specific indications for mastectomy in younger women, which were related either to the presence of oncogenic genetic mutations (BRCA1/2, CHEK2, ATM) or to tumors with unfavorable anatomical location (superomedial or inferomedial quadrants, retroareolar, or extending across the upper quadrants). In such cases, BCS would have resulted in poor cosmetic outcomes, which explained the reason behind the surgical decision of mastectomy with immediate reconstruction.

Probably, the most unexpected finding of this study was the inverse correlation between patient age and the extent of DCIS (r = −0.21). This implies the fact that younger patients could present more regularly with tumors that include an in situ component. While this relationship has not been extensively explored in previous studies, it might introduce another variable in surgical planning and risk stratification, particularly in younger patients.

To account for potential confounding, we performed an exploratory multivariable logistic regression including age, tumor stage, grade, neoadjuvant therapy, and type of surgery. Consistent with the univariate findings, lymphovascular invasion remained the only independent predictor of SLN positivity (OR = 3.42; 95% CI: 2.01–5.83; *p* < 0.001), whereas the other variables were not statistically significant. This reinforces the multifactorial nature of nodal involvement and underscores that classical clinicopathological parameters alone may be insufficient to predict axillary metastasis. These findings are summarized in Table 4.

On the whole, these results underscore the drawbacks of relying exclusively on classical histopathological parameters to guide intraoperative decisions and support the existing need for more complex models (potentially incorporating radiologic, molecular, and even AI-assisted predictors) to optimally design surgical strategies.

### 4.2. Comparison Between Technetium-99m and Indocyanine Green Tracers

The aforementioned results show that both mapping techniques (Tc99m and ICG) performed similarly in terms of sentinel node count and pathological outcomes, with no statistically significant differences between the studied parameters. The fact that some of the *p*-values are far from the significance threshold, combined with the consistency of the results across multiple variables, supports that the selection of one specific tracer does not meaningfully affect nodal assessment. Practically, this implies that oncological surgeons can rely on either tracer method, without any implications for oncologic safety or diagnostic accuracy. The nearly identical average number of excised SLNs and positive nodes emphasizes that the extent of axillary sampling is equivalent regardless of tracer. Nevertheless, the similar rates of lymphatic, vascular, and perineural invasion indicate that tracer choice does not alter the pathological characterization of the disease.

Another advantage (with practical importance) of using ICG is the shorter time required for sentinel node identification. Since ICG is injected directly in the operating room, sentinel node detection can be achieved within a few minutes. In contrast, Tc99m requires the transfer of the patients to the nuclear medicine department. Moreover, the identification process might last 30–40 min or even longer in some cases. This difference emphasizes an additional logistical and procedural benefit of ICG-guided SLNB. Our results support the idea that ICG represents a reliable alternative to Tc99m, especially in centers that aim to avoid the complexity of nuclear medicine scheduling or to reduce costs. On the other hand, Tc99m remains the gold standard, with long-term validation.

Hence, based on our data, the two tracers have equivalent efficacy in terms of sentinel node identification and pathological yield, and the decision between using Tc99m and ICG might reasonably be guided depending on surgeon preference, availability, costs, workflow, rather than by differences in oncological outcomes.

### 4.3. Comparison with the Existing Literature

A network meta-analysis by Mok et al. assessed the conventional and novel SLNB techniques in breast cancer, comparing BD alone or combined with Tc99m to ICG, superparamagnetic iron oxide nanoparticles (SPIO), and contrast-enhanced ultrasound (CEUS). Based on 35 studies, the three methods using Tc99m, ICG, and SPIO showed significantly higher detection rates compared to BD alone. Out of all methods, ICG achieved the lowest rate of false negatives, outperforming both Tc and SPIO. Altogether, the findings point out that ICG and SPIO are not only superior to blue dye alone, but also their performance is equivalent to the standard dual technique of Tc plus blue dye, emphasizing that these novel methods are effective options for SLN mapping in breast cancer [16].

One noninferiority study that evaluated ICG fluorescence versus the standard Tc99m for SLNB in early-stage, clinically node-negative breast cancer patients was the INFLUENCE trial. It involved a total of 102 patients scheduled for BCS and SLNB. Each one of them underwent SLN detection performed using ICG, followed by confirmation with 99mTc. The overall detection rate for SLN was significantly higher using ICG (96.1%) compared to 99mTc (86.4%), while both these methods showed an identical detection rate of pathological SLNs (86.7%). The median value of nodes that were removed was 2, the duration of the procedure was not prolonged, and no adverse events were reported. These findings justify that ICG fluorescence is an efficient, safe, and potentially superior alternative to the radioisotope standard for SLNB in breast cancer [17].

Vermersch et al. performed a randomized controlled trial that assessed SLN detection rate in breast cancer using Tc99m alone versus Tc99m combined with ICG. Totally, 99 patients were included; the SLN identification rate was the primary endpoint. Both methods achieved comparable results: SLN detection rate was 44.0% with dual mapping and 40.8% with Tc99m alone, with no statistically significant difference. Comparing the average values, a higher number of SLNs were identified with dual detection (2.14 vs. 1.77, *p* = 0.09). Most of the nodes were concordant for both tracers, but the ICG method could detect some additional nodes that were missed by Tc99m (13.9%), and conversely (6.5%). Overall, ICG was positive in a higher proportion of cases compared to Tc99m (92.6% vs. 85.2%). No ICG-related adverse events occurred. Their study concludes that dual detection with ICG and Tc99m is sensitive and safe, but not significantly superior to Tc99m alone for successful localization of SLNs [18].

Another study, performed by Agrawal et al., compared techniques of performing SLNB in early breast cancer, evaluating the standard dual method of Tc99m combined with BD against ICG plus BD. A total number of 207 node-negative patients (ranging from T1–T3N0) were included between 2017 and 2019. The overall rate of identification was high (96%), with equivalent performance between Tc99m + BD (95%) and ICG + BD (97%). Both methods detected multiple SLNs in most patients, and the rates of SLN positivity (31.3% vs. 28%) and mean number of metastatic nodes were similar. This study also emphasized a recent decline in the availability of Tc99m, highlighting the practical value of ICG as a reliable option. Therefore, ICG combined with BD has an equivalent diagnostic accuracy to Tc99m + BD and might be particularly appropriate for resource-limited settings [19].

Schaafsma et al. performed a clinical trial that evaluated a hybrid tracer ICG fluorescence with Tc99m radiolabeled nanocolloid for SLNB in breast cancer. A total of 32 patients received periareolar injection of the hybrid tracer preoperatively, followed by lymphoscintigraphy and intraoperative localization using both a γ probe and near-infrared (NIR) imaging. In addition, BD was administered intraoperatively. A total of 48 axillary SLNs were successfully detected with the first two methods, while BD identified only 42 nodes. Fluorescence imaging allowed for the visualization of lymphatic drainage pathways up to 29 h post-injection. The study showed that the combination of radio- and fluorescence-guided tracer is reliable, safe, and enables accurate pre- and intraoperative detection of SLNs, with the advantage of both real-time optical guidance and deep tissue localization [20].

Moreover, a phase II randomized controlled trial conducted by Jung et al. compared SLNB guided by a multimodal method (MMM), combining ICG, radioisotope (RI), and BD versus RI alone in 86 patients, with clinically node-negative breast cancer. Identification of SLNs was successful in all patients across both groups (100%). Despite this, the MMM group had a significantly higher mean number of SLNs that were retrieved (3.4 vs. 2.3, *p* < 0.001). However, detection time was the same between groups. Fluorescent imaging enabled visualization of lymphatic drainage in more than 90% of MMM patients. No adverse reactions or complications occurred. The authors conclude that the MMM approach is safe and enhances SLN when compared to RI alone [21].

In the end, one French study by Guenane et al. compared ICG fluorescence with Tc99m for SLN detection in breast cancer. A total of 40 node-negative patients underwent mapping with preoperative Tc99m injection and intraoperative ICG. Totally, 53 SLNs were excised, all of them being Tc-positive, while 48 (90.6%) were ICG-positive. The 5 non-concordant nodes were Tc-positive but ICG-negative. No adverse effects from ICG were reported. These results show that while ICG fluorescence is considered a safe and popular method for SLN detection, it currently benefits from a slightly lower sensitivity compared to Tc99m and requires operative experience to achieve an equivalent performance [22].

### 4.4. Limitations of the Current Study

The main strength of this study is its prospective design, which allowed for an organized and standardized collection of relevant clinical, surgical, and pathological data for all the included cases. This eliminated the risk of recall bias. Moreover, the study was conducted in a single, high-volume Oncology Institute, where each procedure followed a strict institutional protocol, limiting heterogeneity regarding surgical technique. The relatively large cohort of 476 patients strengthens the statistical power of the analysis and provides a picture of the current clinical practice. In addition, the inclusion of detailed histopathological features (grade, Ki67, TILs, DCIS, lymphovascular/perineural invasion) enabled a detailed exploration of any relations with sentinel node status and surgical margins.

However, several limitations must be acknowledged. To begin with, the study was carried out in a single institution, which may limit the generalizability of the findings across other healthcare settings with different surgical preferences or tracer availability. Furthermore, the choice of tracer was not randomized, but based on institutional availability and the surgeon’s preference, which might cause a selection bias. Even though the overall sample size was sufficient, the number of cases with positive margins (R1) or rare histological subtypes remained relatively small, which could limit the statistical power for performing subgroup analyses. We acknowledge that key SLNB performance metrics, including identification rate, mapping failure rate, and mapping time, were not available in our dataset. This limits direct comparability with other studies and should be considered when interpreting our findings. Lastly, although the study design was prospective, long-term outcomes such as relapse or survival were not evaluated, restricting the conclusions to short-term surgical and pathological endpoints.

Overall, the strengths of the study consist of its prospective design, systematic methodology, and large sample size, whereas its main limitations are its single-center, non-randomized design and the absence of long-term oncological outcomes.

## 5. Conclusions

All in all, Tc99m and ICG demonstrated no statistically significant differences in sentinel lymph node detection or pathological yield. Both tracers provided accurate axillary staging and yielded comparable oncological outcomes. Beyond their equivalence regarding the diagnostic performance, ICG brings a few practical advantages, including no necessity for nuclear medicine infrastructure and increased intraoperative workflow. Collectively, these findings justify why ICG is a safe, efficient, and feasible alternative to Tc99m for SLN mapping in breast cancer. However, further randomized studies (with a high number of included subjects) are required to confirm these results and to investigate the applicability of ICG in routine surgical practice and its cost-effectiveness.

## Figures and Tables

**Table 1:** Demographic and surgical characteristics of the included patients.

Parameter	Value
Mean age (SD)	59.39 (10.96)
Median age (IQR)	60 (52–69)
Neoadjuvant treatment—Yes	130 (27.31%)
Neoadjuvant treatment—No	346 (72.69%)
Breast-conserving surgery	371 (77.94%)
Simple mastectomy	105 (22.06%)

**Table 2:** Characterization of the sentinel nodes by tracer.

Tracer	ICG	Tc99m
Mean of removed nodes	3.20	3.23
SD of removed nodes	1.51	2.25
Minimum number of removed nodes	1	1
Maximum number of removed nodes	8	11
Mean of positive nodes	0.36	0.35
SD of positive nodes	0.71	0.74
Minimum number of positive nodes	0	0
Maximum number of positive nodes	4	5

**Table 3:** Histopathological characteristics of the included patients.

Parameter	Value/Type
Most common histology	NST/invasive ductal
Nottingham grade 1	172
Nottingham grade 2	250
Nottingham grade 3	54
Ki67–mean (SD)	17.7 (11.9)
Ki67–median (IQR)	15 (10–25)
TILs–mean (SD)	7.5 (10.9)
TILs–median (IQR)	5 (1–10)

**Table 4:** Multivariable logistic regression for predictors of SLN positivity.

Variable	Odds Ratio (OR)	95% CI	*p*-Value
Age (continuous)	0.99	0.97–1.01	0.320
Tumor stage (T)	1.25	0.92–1.68	0.150
Tumor grade	1.12	0.85–1.48	0.420
Neoadjuvant therapy	1.08	0.71–1.65	0.720
Type of surgery (Mastectomy vs. BCS)	0.95	0.61–1.48	0.810
Lymphovascular invasion (yes vs. no)	3.42	2.01–5.83	<0.001

## Data Availability

Data are available upon reasonable request from the corresponding author.

## Abbreviations

The following abbreviations are used in this manuscript:

SLNB	Sentinel Lymph Node Biopsy
RI	Radioisotope
BD	Blue Dye
ICG	Indocyanine Green
SPIO	Superparamagnetic Iron Oxide
FDA	Food and Drug Administration
SLN	Sentinel Lymph Node
Tc99m	Technetium-99m
IOCN	Institute of Oncology “Prof. Dr. I. Chiricuță” (Cluj-Napoca, Romania)
SD	Standard Deviation
IQR	Interquartile Range
BCS	Breast-Conserving Surgery
NST	No Special Type (Invasive carcinoma of no special type)
DCIS	Ductal Carcinoma In Situ
TILs	Tumor-Infiltrating Lymphocytes
R0	Negative Surgical Margins
R1	Positive Surgical Margins
ITC	Isolated Tumor Cells
Nx	Nodal Status Undetermined
MMM	Multimodal Method
